# Parents’ Speech in the NICU and Language Development of Very Preterm Children at 12 and 24 Months

**DOI:** 10.1016/j.jpedcp.2025.200156

**Published:** 2025-06-12

**Authors:** Anette Aija, Eva Ståhlberg-Forsén, Liis Toome, Laura Aarnos, Sari Ahlqvist-Björkroth, Suvi Stolt, Liisa Lehtonen

**Affiliations:** 1 University of Turku, Turku, Finland; 2 Department of Neonatal and Infant Medicine, Tallinn Children's Hospital, Tallinn, Estonia; 3 Department of Speech-Language Pathology, University of Helsinki, Helsinki, Finland; 4 Speech and Language Pathology, Åbo Akademi University, Turku, Finland; 5 Department of Psychology and Speech-Language Pathology, University of Turku, Turku, Finland; 6 Department of Paediatrics and Adolescent Medicine, Turku University Hospital, Turku, Finland

**Keywords:** language environment analysis, parental speech, language development

## Abstract

**Objective:**

It is unclear if speech input in neonatal units improves delayed language development in very preterm infants. This longitudinal study investigated whether the parents’ speech in family-centered neonatal units associated with language outcomes in children born very preterm.

**Study design:**

The auditory environment of 82 infants born <32 gestational weeks was recorded using Language Environment Analysis at 32–34 weeks of postmenstrual age. The language environment was analyzed for the total recording time and the periods when the parents were present. Receptive and expressive language skills were measured at 1 year (MacArthur-Bates Communicative Development Inventory) and 2 years of corrected age (Reynell Developmental Language Scales III).

**Results:**

Father's word frequency on recording day (*b* ln-scale 0.05, 95% CI 0.003–0.09, *P* = .04), and conversational turns with the mother during 14 days (*b* ln-scale 0.08, 95% CI 0.01–0.16, *P* = .03) were positively associated with expressive lexicon size at 1 year. Overall adult word frequency was negatively associated with the child's expressive language skills at 2 years of corrected age (*b* in-scale −0.13, 95% CI -0.24–-0.01, *P* = .03).

**Conclusions:**

Parents’ speech in the neonatal unit may support language development of children born preterm, whereas high total adult words - including the time when parents were not present in the unit - may impair it. Our findings should be cautiously interpreted as the associations were weak.

**Trial registration:**

Auditory Environment by Parents of Preterm Infants (APPLE), registration number: NCT04826978, date of registration: 2021-03-29.

Preterm infants are at an increased risk for various neurodevelopmental problems, including deficits and delays in early lexical abilities and, therefore, difficulties in later language development.^1^, ^2^, ^3^, ^4^, ^5^, ^6^, ^7^ While the specific mechanism that leads to poor developmental outcomes in preterm infants remains unclear, it is probably due to perinatal (eg, gestational age at birth), demographic (eg, the family's socioeconomic status, maternal education), health-related, and neonatal environmental factors.^5^ The mother's womb protects the fetus from harmful factors that could compromise the developing auditory system.^8^ After a preterm birth, infants experience an abrupt change from the intrauterine sound environment to that of the neonatal intensive care unit (NICU). A recent study that quantitatively compared the auditory exposures of fetuses and preterm infants found that preterm infants in the NICU are exposed to five times less adult speech than fetuses.^9^ Studies conducted in the natural home environment with normally developing children have highlighted the importance of language input in supporting healthy child language development.^10^^,^^11^ In the NICU environment, language deprivation could be categorized into 2 scenarios, “too quiet” or “too noisy”.^12^ The language development of preterm infants may be impaired by exposure to noise and high sound levels, which are common in the NICU environments.^13^, ^14^, ^15^

Involving parents in the neonatal care of their preterm infants may enhance better language development outcomes. A study by Caskey et al has found a positive impact of adult speech in the NICU on preterm infants' later cognitive and language development at 18 months of corrected age.^16^ A randomized, controlled, parent-driven language enrichment study in the NICU found that an increase in parent-infant conversational turns and adult word count was positively associated with improved language scores at two years of corrected age.^17^ However, Stahlberg-Forsén et al have reported that a high number of overheard adult words near the preterm infant is negatively associated with auditory lexical processing accuracy at 18 months of corrected age.^18^ The existing research on these aspects of preterm infants’ language development remains limited and partially controversial. It is unclear whether the better language development outcomes are due to overall speech heard in the NICU, due to interactive and infant-directed parental speech, and whether there are any differences in the impact of maternal and paternal input.

The objective of this study was to investigate the associations between mothers' and fathers’ speech and parent-infant conversational turns during neonatal care and later language outcomes of children born very preterm. We hypothesized that higher exposure to parental speech and parent-infant conversational turns during neonatal care associate with larger receptive and expressive lexicon size at 1 year of corrected age and better receptive and expressive language skills at 2 years of corrected age.

## Methods

### Participants

The APPLE Study (Auditory environment by Parents of Preterm infant; Language development and Eye-movements) is a longitudinal study conducted in 2 neonatal units located in Turku, Finland, and Tallinn, Estonia. The Ethics Committee, Hospital District of Southwest Finland, and the Research Ethics Committee of the University of Tartu, Estonia have confirmed the study protocol. Preterm infants born before 32 gestational weeks and their parents, speaking Finnish, Swedish, Estonian, or Russian, were recruited in the APPLE study. The exclusion criteria were multiple pregnancies of more than 2 fetuses, life-threatening diseases, major congenital anomalies, chromosomal anomalies, and syndromes of clinical significance. Recruitment occurred from February 2017 through December 2020, and the participants were monitored until the corrected age of 2 years. A total of 82 infants were included in this subanalysis, including families speaking mainly (70% of the time) Finnish or Estonian around the child (Figure. Flow chart).

**Figure:**
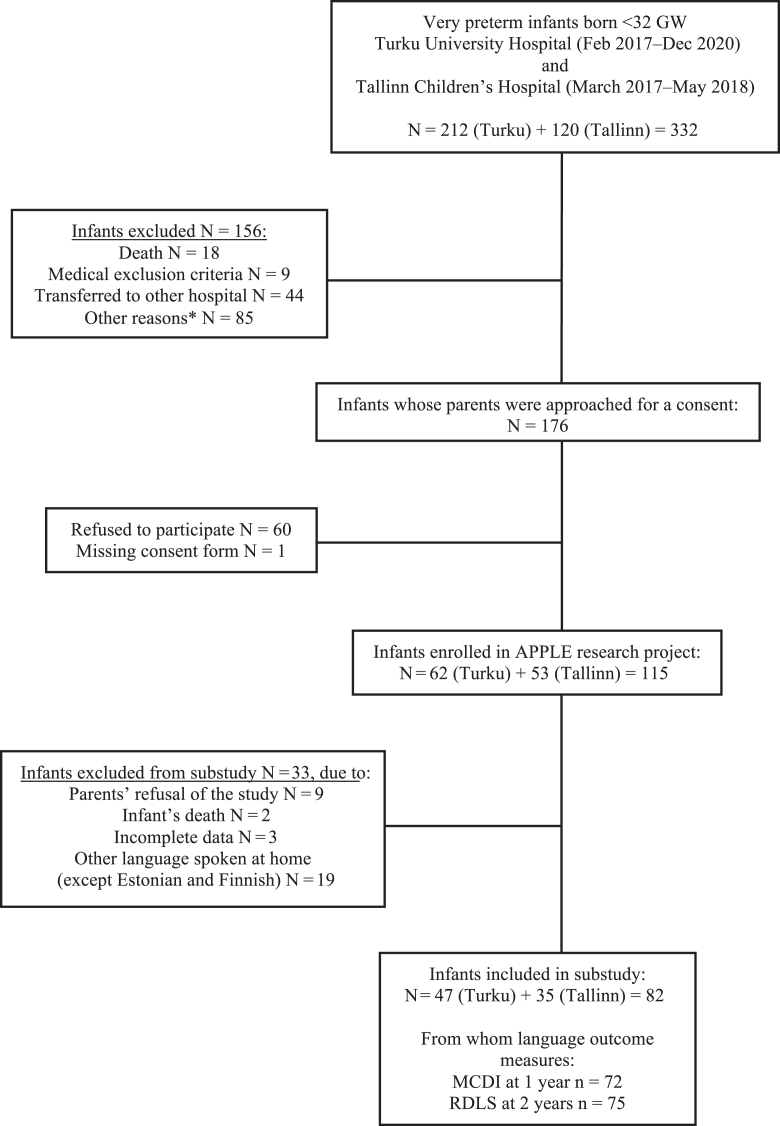
The flow chart presents the recruitment process of APPLE research project and additional exclusion criteria of this substudy. ∗Other reasons to exclude infants from the study included license and technical issues, social reasons, native language, and multiple pregnancy of more than 2 fetuses. *GW*, gestational week.

Written informed consent form was obtained from the parents of the preterm infants, and infant and family characteristics were collected from hospital records and the parents' questionnaire. Participating mothers and fathers filled in the Closeness Diary ^19^ for 14 days when their infant was at 32–34 weeks of postmenstrual age, including information about the parent's presence in the unit, parent-infant skin-to-skin contact and holding the infant, each reported with a 5-minute resolution. Finnish growth references were used to calculate birth weight z-scores for both study sites. All patients in Tallinn and all patients born before 30 gestational week in Turku were screened for retinopathy of prematurity, subject to the local guidelines.

### Measurements

#### Auditory Environment in the NICU

The infants’ auditory environment was measured based on a 16-hour recording at 32–34 weeks of postmenstrual age using Language Environment Analysis (LENA Organization). The LENA analysis automatically estimates female and male adult word count (AWC), conversational turn count (CTC), child vocalization count (CVC), and the duration of silence, TV/electronic sounds, and noise. Conversational turns are exchanges between the child and an adult within five seconds. LENA defines child vocalizations as speech-related communicative sounds produced by the child, surrounded by 300 milliseconds of silence or nonspeech, and excluding vegetative sounds and cries. The LENA microphone was kept near the infant – within 10 cm from the head when staying in the cot or incubator and within 30 cm during holding or skin-to-skin contact with a parent. The validity of the LENA system in Finnish and Estonian languages was tested using this study material from both study units. The LENA data validity was compared to native language speaking coders who listened and categorized the audio data. This study confirmed the validity of the AWC provided by LENA. The best validity was found for female words. However, the validity for CTC and CVC was modest or low.^20^

We used LENA data from the periods when the parents were present during the time interval between 7 AM and 10 PM, assuming that the parents would be awake and potentially verbally communicating with the infant. In the *posthoc* analysis, overall adult word frequency included all AWC between 7 AM and 10 PM, including periods when parents were absent. The parent's presence in the NICU was determined from the closeness diaries. The mother's and father's word frequencies (words/h) were collected from the LENA data using female and male word counts. CTC and CVC were collected from the LENA data using time intervals when each parent was present in the NICU. In further analysis, the exposure measurements were based on the frequency measurements (words or count per hour) when the parent was present during the recording day and the information on the parents' presence for 2 weeks. For example, exposure to the parent speech = [total number of words when the parent was present during the analyzed recording (count)/total time the parent was present during the analyzed recording (hours)] x the parent's presence (hours) for 14 days.

In 3 cases, the mother was a single parent. Additionally, 4 fathers in Turku and 12 fathers in Tallinn were not present during the recording day, so even though they visited their infants on other days during the NICU stay, the fathers' word frequency, CTC, and exposure for 14 days were excluded from the analysis. In instances where a parent was absent during the recording day of one of the twins, the word frequency recorded during the other twin's session was used.

#### Language Development

Information on the infant's lexical abilities at 12 months of corrected age was gathered using Finnish and Estonian versions of the parental report method MacArthur-Bates Communicative Development Inventory (MCDI).^21^^,^^22^ The number of words in the word lists included in the Finnish MCDI is 379 words, and in the Estonian version, the respective number is 386 words. The parents reported how many words in these word lists the child comprehended or expressed at 1 year of the corrected age. The number of words reported by the parent was converted into percentages for the statistical analysis based on the total number of words in the word lists.

Information on the child's general language development (receptive and expressive language ability) was acquired through testing at 2 years of corrected age using the Finnish version of the Reynell Developmental Language Scales third edition (RDLS).^23^ The translated Finnish version of the RDLS was used for the Estonian data. The raw scores for the RDLS were used since the RDLS has not been standardized for Estonian.

#### Statistical Analyses

Descriptive statistics were used to describe the family, infant, and recording day characteristics, the parent's presence, and the LENA measures (parent word frequency, conversational turn frequency, child vocalization frequency). The environmental contexts of the study sites were compared using Fisher exact or Wilcoxon rank sum tests depending on the data type. The associations between the language outcome measures (receptive and expressive scores of MCDI and RDLS as dependent variables) and the LENA measures (adult word frequency, parent word frequency, conversational turn frequency, and child vocalization frequency as independent variables) were assessed using separate mixed linear models with the hospital and twin pair as random factors. All models were adjusted for gestational age (GA), sex, twin status, and parent education. The natural logarithms of both the dependent and independent variables were used to fulfill the linear model assumptions. Statistical assumptions were tested, defined by a significance with a *P* value < .05. The Stata 16.0 software (StataCorp LLC) was used for the statistical analyses.

## Results

### Background of the Study Population

A total of 82 preterm infants and their families (82 mothers and 79 fathers) were included in the analysis. The infant and family characteristics are described in Table I. We recruited patients from 2 centers that had both implemented family-centered care practices. However, the NICU environment was different at the 2 study sites. During the LENA recording day, families in Turku were mostly (57%) treated in single-family rooms, whereas in Tallinn most infants (91%) shared the room with at least one other patient. All patients in Turku were cared for on an open bed with a thermal mattress, whereas 49% of the patients in Tallinn were treated in an incubator during the recording day. All infants at both sites needed a feeding tube to support enteral feeding. The environmental context during the recording day is presented in Table II. The LENA recordings were performed at a mean age of 33^2/7^ weeks of postmenstrual age (SD 4 days) at both study sites. Table III presents the descriptive statistics for parental presence and the LENA measures during the recording day, exposure for 14 days, and the language outcomes at 1 and 2 years of corrected age.

**Table I:** Infant and family characteristics

Characteristic	n = 82
Gestational age, wks	28 (2)
Birth weight, g	1148 (373)
Male sex	44 (54)
Cesarean section	48 (59)
Twin	28 (34)
BPD diagnosis at 36 wks PMA	38 (46)
IVH Grade III or IV, or PVL	1 (1.2)
Treated ROP	3 (4)
Operated NEC	2 (2.4)
Positive blood culture sepsis	10 (12)
Hearing status, pathologic	2 (2.4)
Hearing aid needed at 1 y of corrected age	0
Maternal age, y	31.3 (6)
Paternal age, y	33.7 (6)
Maternal education level	
Basic education	4 (5)
Upper secondary school or vocational education and training	47 (57)
University	31 (38)
Paternal education level	
Basic education	5 (6)
Upper secondary school or vocational education and training	58 (71)
University	15 (18)
Unknown	4 (5)
Sibling living at home	45 (55)
Previous child in the NICU	4 (5)
Change in family status during first 2 y	7 (9)
Other languages spoken in the family (<30% of the time)	3 (4)
Neurological diagnosis∗ at 2 y of age	7 (9)
Attending in kindergarten	31 (38)
Average age starting kindergarten, mo (min; max)	20 (12; 28)

*BPD,* bronchopulmonary dysplasia; *IVH,* intraventricular hemorrhage; *NEC,* necrotizing enterocolitis; *NICU,* neonatal intensive care unit; *PMA,* postmenstrual age; *PVL,* periventricular leukomalacia; *ROP,* retinopathy of prematurity.

Data are presented as n (%) or mean (SD).

∗ Visual, hearing, developmental, or motor impairment.

**Table II:** Environmental context during the recording day

	Turku (n = 47)	Tallinn (n = 35)	*P* value
Patients per room			**<.001**
1	27 (57)	3 (9)	
2	20 (43)	16 (46)	
3	0 (0)	4 (11)	
4	0 (0)	12 (34)	
Temperature control			**<.001**
Incubator	0 (0)	17 (49)	
Thermal mattress	17 (36)	12 (34)	
None	30 (64)	6 (17)	
Breathing support			.19
Invasive ventilation	5 (11)	0 (0)	
CPAP/NIV-NAVA	6 (13)	2 (6)	
CPAP/High flow nasal cannula alternating	2 (4)	1 (3)	
High flow nasal cannula	17 (36)	14 (40)	
None	17 (36)	18 (51)	

*CPAP,* continuous positive airway pressure; *NIV-NAVA,* noninvasive neurally adjusted ventilatory assist.

Data are shown as absolute numbers (percentage).

Bolded *P* values indicate statistical significance (*P* < .05).

**Table III:** Descriptive statistics for parental presence from the parental Closeness Diaries, LENA measures from the LENA recordings in the unit, and language measures from MacArthur-Bates Communicative Development Inventory measured at 1 y of corrected age, and Reynell Developmental Language Scales measured at 2 y of corrected age

Measures	n = 82	
	Median	Min–Max
During the recording day		

Mother's presence in the NICU (h)	8.0	0.8-13.8
Father's presence in the NICU (h)	2.5	0-13.7
Mother's word frequency (words/h)	615	0.4-3591
Father's word frequency (words/h)	170	0-2291
Conversational turn frequency when the mother was visiting (count/h)	5	0-77
Conversational turn frequency when the father was visiting (count/h)	4	0-107
Child vocalization frequency when the mother was visiting (count/h)	21	2-204
Child vocalization frequency when the father was visiting (count/h)	13	0-237

Exposure for 14 days		

Mother's presence in the NICU (h)	116	43-210
Father's presence in the NICU (h)	40	0-153
Exposure to mother's speech (words)	69594	76-283115
Exposure to father's speech (words)	7075	0-101101
Conversational turns count (CTC) when the mother was visiting	555	0-4314
CTC when the father was visiting	186	0-3514
Child vocalizations count (CVC) when the mother was visiting	2415	289-39194
CVC when the father was visiting	669	0-7744

Language outcome∗		

MCDI† receptive	33	1-177
MCDI† expressive	2.5	0-31
RDLS‡ receptive	13	0-39
RDLS‡ expressive	3	0-19

*LENA,* Language Environment Analysis; *MCDI,* MacArthur-Bates Communicative Development Inventory (receptive and expressive lexicon) measured at 1 y of corrected age; *NICU,* neonatal intensive care unit; *RDLS,* Reynell Developmental Language Scales (receptive and expressive language score) measured at 2 y of corrected age.

∗ MCDI n = 72, RDLS n = 75.

† In the norming sample of the Finnish version of the MacArthur-Bates Communicative Development Inventory, the descriptive statistics at 1,0 are for the receptive words: Median 67, Min-Max 6–263; for the expressive words: Median 3,5, Min-Max 0–60.

‡ raw scores used; in the norming sample of the Finnish version of the Reynell Developmental Language Scales III, the mean value and the typical variation (-1SD–+1SD) of the raw scores at 2 y of age for the receptive scale are 20 (12–28) and for the expressive scale 9 (4 –14).

### Association Between the Words Heard in the NICU and Language Development

Father's word frequency per hour (*b* in ln-scale 0.05, 95% CI 0.003–0.09, *P* = .04) was positively associated with expressive lexicon size at one year of corrected age. No significant associations were found between the same measures using the mother's words and the child's language development. In *posthoc* analysis, where we included all adult words measured during the recording day between 7 am and 10 pm, also when the parents were not present, the overall adult word frequency was negatively associated with the child's expressive language skills at two years of corrected age (*b* in ln-scale −0.13, 95% CI -0.24– −0.01, *P* = .03). Adjusted separate linear mixed models are presented in Table IV.

**Table IV:** Associations are presented between LENA measures (expressed in natural logarithm-scale) and language outcome measures (expressed in natural logarithm-scale). Statistics for the separate mixed linear models are presented∗

	Coefficient in ln-scale	*P* value	95% CI
Overall adult word frequency (words/h)			
MCDI receptive	−0.03	.47	−0.11-0.05
MCDI expressive	0.02	.59	−0.06-0.10
RDLS receptive	0.01	.94	−0.14-0.15
RDLS expressive	−0.13	**.03**	−0.24-0.01
Mother's word frequency (words/h)			
MCDI receptive	−0.05	.14	−0.12-0.02
MCDI expressive	−0.01	.81	−0.08-0.07
RDLS receptive	−0.01	.84	−0.14-0.12
RDLS expressive	−0.06	.28	−0.18-0.05
Father's word frequency (words/h)			
MCDI receptive	−0.03	.23	−0.08-0.02
MCDI expressive	0.05	**.04**	0.003-0.09
RDLS receptive	0.03	.59	−0.08-0.13
RDLS expressive	−0.01	.87	−0.09-0.08
Conversational turns when mother present (count/h)			
MCDI receptive	0.05	.59	−0.12-0.21
MCDI expressive	0.10	.12	−0.03-0.23
RDLS receptive	−0.02	.89	−0.23-0.20
RDLS expressive	0.04	.75	−0.19-0.27
Conversational turns when father present (count/h)			
MCDI receptive	0.14	.14	−0.05-0.32
MCDI expressive	0.05	.47	−0.08-0.18
RDLS receptive	−0.001	.99	−0.23-0.23
RDLS expressive	0.04	.73	−0.19-0.27
Child vocalizations when mother present (count/h)			
MCDI receptive	0.01	.86	−0.14-0.17
MCDI expressive	0.04	.47	−0.07-0.16
RDLS receptive	−0.02	.84	−0.21-0.17
RDLS expressive	0.06	.55	−0.14-0.26
Child vocalizations when father present (count/h)			
MCDI receptive	0.11	.16	−0.04-0.26
MCDI expressive	0.08	.21	−0.04-0.20
RDLS receptive	−0.01	.97	−0.24-0.23
RDLS expressive	0.07	.52	−0.14-0.28
Exposure to mother's words for 14 d			
MCDI receptive	−0.05	.15	−0.12-0.02
MCDI expressive	0.01	.79	−0.07-0.09
RDLS receptive	−0.01	.90	−0.15-0.13
RDLS expressive	−0.05	.39	−0.17-0.07
Exposure to father's words for 14 d			
MCDI receptive	−0.02	.10	−0.05-0.004
MCDI expressive	0.03	.05	−0.0002-0.06
RDLS receptive	0.03	.43	−0.04-0.10
RDLS expressive	0.005	.86	−0.05-0.06
Conversational turns when mother present for 14 d			
MCDI receptive	0.03	.51	−0.06-0.11
MCDI expressive	0.08	**.03**	0.01-0.16
RDLS receptive	−0.01	.86	−0.16-0.13
RDLS expressive	−0.05	.44	−0.18-0.08
Conversational turns when father present for 14 d			
MCDI receptive	−0.004	.93	−0.10-0.09
MCDI expressive	0.01	.83	−0.07-0.09
RDLS receptive	0.03	.67	−0.09-0.14
RDLS expressive	0.06	.35	−0.06-0.18
Child vocalizations when mother present for 14 days			
MCDI receptive	0.03	.66	−0.12-0.19
MCDI expressive	0.08	.16	−0.03-0.20
RDLS receptive	−0.01	.94	−0.20-0.18
RDLS expressive	0.11	.29	−0.09-0.31
Child vocalizations when father present for 14 d			
MCDI receptive	0.04	.54	−0.08-0.15
MCDI expressive	0.03	.50	−0.06-0.12
RDLS receptive	−0.004	.96	−0.16-0.15
RDLS expressive	0.07	.37	−0.08-0.22

*MCDI,* MacArthur-Bates Communicative Development Inventory (receptive and expressive lexicon) measured at 1 y of corrected age; *RDLS,* Reynell Developmental Language Scales (receptive and expressive language score) measured at 2 y of corrected age.

MCDI with mother variable n = 72, RDLS with mother variable n = 75.

MCDI with father variable n = 60; RDLS with father variable n = 58.

Statistical significance when *P* < .05, marked in bold text.

∗ Fixed effects: gestational age, sex, twin status, parent's education; Random effect: twin pair, hospital.

### Association Between CTC in the NICU and Language Development

A higher CTC during mother's presence within 14 days was positively associated with larger expressive lexicon size at one year of corrected age (*b* in ln-scale 0.08, 95% CI 0.01–0.16, *P* = .03) in linear mixed models adjusted for background factors. CTC during father's presence was not associated with the lexicon size at 1 year of corrected age nor with language outcome measures at 2 years of corrected age (Table IV).

### Background Factors and Language Development

In all adjusted models, the MCDI receptive and expressive scores were positively associated with GA (*P* ranged from .001 to .02). RDLS receptive and expressive scores were associated with sex (*P* ranged from <.001 to .03), girls had higher scores than boys. Twin status as a covariate was negatively associated with RDLS receptive and expressive scores (*P* ranged from .001 to .02). The parent's education as a covariate was not associated with the outcome variables.

## Discussion

The main finding of our study was that if the infant had heard more father's words in the NICU, his/her expressive lexicon size was larger at one year of corrected age. Additionally, a higher CTC during mother's presence was associated with larger expressive lexicon size at one year of corrected age. In the *posthoc* analyses, which included all adult word input regardless of parental presence, a negative association was observed between the total word count and the child's expressive language skills at two years of corrected age.

Recent meta-analyses have shown that parental linguistic input is a key environmental factor and essential for children's language development.^24^ Studies involving healthy toddlers have additionally highlighted the importance of paternal language input in fostering advanced child communicative skills and language production, complementing the influence of overall speech input.^25^^,^^26^ Several studies have reported that fathers produce a significantly smaller proportion of words and parentese than mothers.^25^^,^^27^ Shapiro et al have reported in their study, including healthy full-term infants, that paternal language input was about half of the maternal language input, whereas paternal parentese predicted more child vocalizations.^27^ In our study, the average word frequency per hour during the father's presence was less than one third of the word frequency during the mother's presence. Furthermore, the estimated amount of fathers' words over 2 weeks, accounting for father's presence, was 10% of the amount of mothers' words. Despite the quantitatively lower levels of paternal presence and speech input in the NICU compared to maternal contributions, fathers' words seem to predict better lexical development for preterm infants, although the associations were weak. Overall, father involvement in different contexts and cultures has been shown to have long-term positive effects on child development. The roles of fathers and mothers appear to be complementary, providing different experiences for children.^28^ Even though the fathers' overall contribution was less than that of the mothers', they may have enriched the language environment of the infants. Furthermore, the early involvement of fathers during NICU care may support their later involvement in child care, which may also be the mechanism behind this effect. These findings highlight the importance of promoting paternal involvement in the NICU.

Several studies have shown that interactive adult-child 2-way conversations are meaningful in healthy language development in typically developing children.^11^^,^^29^ In the preterm population, parents' speech rate and maternal interactive features were associated with better language scores.^30^ Our findings showed that CTC during mother's presence was associated with better expressive language development at one year of corrected age in our study population of very preterm infants. We acknowledge that our effect sizes were small, and effects were not robust as modifications of the statistical models affected the statistical significance of the results. Even though many questions remain, infant-directed speech seems to influence language development in children born preterm.^30^ Additional studies are needed to examine the potential mechanisms behind the effects of maternal and paternal language input, infant-directed speech, and their influence on preterm infant language development.

Previous research has shown that integrating the parents into neonatal care has a positive impact on later child development.^31^, ^32^, ^33^, ^34^ We included patients from two different units, both of which had integrated parents in the infant care. However, the units differed in their architecture, and only one of them had implemented structured training for the staff to develop their skills to collaborate with parents. The parents' word frequency and parent-infant conversational turn frequency were significantly higher in the unit where there were mainly single-family rooms and the staff had received the Close Collaboration with Parents training.^35^ We know that the Close Collaboration with Parents intervention increases the parents' presence^36^ and the quality of family-centered care in the NICU.^37^^,^^38^ Although many units use family-centered care principles, promoting the infant's communication and linguistic development may be overlooked. Our study was done in a realistic environment, without targeted interventions to promote the parents' speech or vocal communication. It has been shown that an infant-directed reading intervention in the hospital environment predicted more conversational turns between the parent and the infant by 36 weeks of postmenstrual age.^39^ A parent-driven language intervention in the NICU increased adult word count at 36 weeks of postmenstrual age and was associated with improved language scores in children born preterm at 2 years of corrected age.^17^ Parents who are trained to speak with their infant may continue this pattern after discharge, which could have an impact on the long-term developmental outcomes of preterm infants. Thus, training the staff is also one possible tool to increase the parents' communication with their infant.

Our earlier study by Ståhlberg-Forsén et al, which included a subpopulation of this study (Finnish-speaking families from one unit), reported that a high number of overheard adult words near the infant during neonatal care was negatively associated with lexical processing accuracy at 18 months of corrected age.^18^ This larger study population, including preterm infants from 2 countries and families speaking either Finnish or Estonian, confirmed the finding that the total amount of adult words was negatively associated with the child's expressive language skills at 2 years of corrected age. There is a balance between developmentally supportive language input and asynchronous talk that can be regarded as noise. A recent review identified people congregations as the primary source of noise in the NICUs.^15^ Our findings support previous recommendations emphasizing synchronous, sensitive, infant-directed talk,^12^ as this seems more meaningful than asynchronous adult talk in the neonatal unit. Encouraging parental presence in the NICU may facilitate infant-directed and soft speech, which potentially benefits subsequent language development.

The strengths of this study are the observational measures of speech in real-life situations, information on parents’ presence over an expanded time window utilizing diary data, and data from two NICUs to expand the variation. The long follow-up period is also a strength. GA, sex, and plurality as covariates associated with the outcome variables, consistently with earlier studies, showing the validity of the statistical models.

This study has limitations. Overall, we report the significant findings with precaution as our analysis involved multiple comparisons and modification of the statistical model, eg, adding twins as a random effect, affected the statistical significance of the results. LENA validation study was done both in Finnish and Estonian languages using our study population showing that there was a high agreement between LENA and human estimates on adult and female words, whereas male words, child vocalizations, and conversational turns had only moderate agreement, indicating the limits of this automatic measuring.^20^ Although we did not observe a performance bias, it is possible that some parents may have been influenced by the awareness of being recorded. Based on the diary data, we analyzed the time periods when a parent(s) was present. However, we do not know how much parents participated in the recorded discussions and whether the speech was between parent-staff, parent-infant or staff-infant. Another element of potential bias is that the parents were self-reporting their presence in the NICU. Coincidentally, 19% of the fathers were not in the NICU during the recording day, due to which they were excluded from the analyses, which may have influenced the outcome effect. The language outcomes could have been also influenced by the later language exposures in the home environment. In such case, the early exposures can serve as markers rather than causal factors. Future studies are needed to investigate further the effects of early NICU environment on later child development.

## Conclusion

The study found that both mothers' and fathers' speech during neonatal hospital care in the neonatal unit supported language development in children born preterm, whereas overheard adult words impaired it. We report our findings with precaution, as the effect sizes were small. Future studies are needed to investigate the importance of parental speech and the quality of speech in relation to the child's language development. It is important to optimize the auditory NICU environment for preterm infants.

## CRediT authorship contribution statement

**Anette Aija:** Writing – review & editing, Writing – original draft, Visualization, Resources, Methodology, Investigation, Formal analysis, Data curation, Conceptualization. **Eva Ståhlberg-Forsén:** Writing – review & editing, Methodology, Investigation, Conceptualization. **Liis Toome:** Writing – review & editing, Supervision, Resources, Project administration, Methodology, Investigation, Data curation, Conceptualization. **Laura Aarnos:** Writing – review & editing, Data curation. **Sari Ahlqvist-Björkroth:** Writing – review & editing, Resources, Methodology, Investigation, Formal analysis, Data curation, Conceptualization. **Suvi Stolt:** Writing – review & editing, Resources, Project administration, Methodology, Investigation, Funding acquisition, Conceptualization. **Liisa Lehtonen:** Writing – review & editing, Writing – original draft, Visualization, Validation, Supervision, Resources, Project administration, Methodology, Investigation, Funding acquisition, Formal analysis, Data curation, Conceptualization.

## Data Statement

Data sharing statement available at www.jpeds.com.

## Declaration of Competing Interest

This work was supported by the 10.13039/501100004325Signe and Ane Gyllenberg Foundation and the Finnish Pediatric Research Foundation. The authors have no conflicts of interest to disclose.

## Supplementary Data

Data Statement

## References

[1] Stolt S., Haataja L., Lapinleimu H., Lehtonen L. (2009). The early lexical development and its predictive value to language skills at 2 years in very-low-birth-weight children. J Commun Disord.

[2] Barre N., Morgan A., Doyle L.W., Anderson P.J. (2011). Language abilities in children who were very preterm and/or very low birth weight: a meta-analysis. J Pediatr.

[3] van Noort-van der Spek I.L., Franken M.C.J.P., Weisglas-Kuperus N. (2012). Language functions in preterm-born children: a systematic review and meta-analysis. Pediatrics.

[4] Stolt S., Mäkilä A.M., Matomäki J., Lehtonen L., Lapinleimu H., Haataja L. (2014). The development and predictive value of gestures in very-low-birth-weight children: a longitudinal study. Int J Speech Lang Pathol.

[5] Zimmerman E. (2018). Do infants born very premature and who have very low birth weight Catch up with their full term Peers in their language abilities by early School age?. J Speech Lang Hear Res JSLHR.

[6] Sanchez K., Spittle A.J., Cheong J.L., Thompson D.K., Doyle L.W., Anderson P.J. (2019). Language in 2-year-old children born preterm and term: a cohort study. Arch Dis Child.

[7] Joensuu E., Munck P., Setänen S., Lipsanen J., Huhtala M., Lapinleimu H. (2021). Associations between language at 2 Years and Literacy skills at 7 Years in preterm children born at very early gestational age and/or with very low birth weight. Child Basel Switz.

[8] McMahon E., Wintermark P., Lahav A. (2012). Auditory brain development in premature infants: the importance of early experience. Ann N Y Acad Sci.

[9] Monson B.B., Ambrose S.E., Gaede C., Rollo D. (2023). Language exposure for preterm infants is Reduced relative to fetuses. J Pediatr.

[10] Huttenlocher J. (1998). Language input and language growth. Prev Med.

[11] Zimmerman F.J., Gilkerson J., Richards J.A., Christakis D.A., Xu D., Gray S. (2009). Teaching by listening: the importance of adult-child conversations to language development. Pediatrics.

[12] Rand K., Lahav A. (2014). Impact of the NICU environment on language deprivation in preterm infants. Acta Paediatr Oslo Nor.

[13] Best K., Bogossian F., New K. (2018). Language exposure of preterm infants in the neonatal Unit: a systematic review. Neonatology.

[14] Liszka L., Smith J., Mathur A., Schlaggar B.L., Colditz G., Pineda R. (2019). Differences in early auditory exposure across neonatal environments. Early Hum Dev.

[15] Andy L., Fan H., Valerie S., Jing W. (2025). Systematic review of environmental noise in neonatal intensive care units. Acta Paediatr Oslo Nor.

[16] Caskey M., Stephens B., Tucker R., Vohr B. (2014). Adult talk in the NICU with preterm infants and developmental outcomes. Pediatrics.

[17] McGowan E.C., Caskey M., Tucker R., Vohr B.R. (2023). A randomized controlled trial of a neonatal intensive care Unit language intervention for parents of preterm infants and 2-year language outcomes. J Pediatr.

[18] Ståhlberg-Forsén E., Latva R., Aija A., Lehtonen L., Stolt S. (2022). Language environment and parent-infant close contact in neonatal care and emerging lexical abilities of very preterm children-a longitudinal study. Acta Paediatr.

[19] Axelin A., Raiskila S., Lehtonen L. (2020). The development of data collection tools to measure parent-infant closeness and family-centered care in NICUs. Worldviews Evid Based Nurs.

[20] Ståhlberg-Forsén E., Aija A., Kaasik B., Latva R., Ahlqvist-Björkroth S., Toome L. (2021). The validity of the Language Environment Analysis system in two neonatal intensive care units. Acta Paediatr Oslo Nor.

[21] Lyytinen P. (1999). Varhaisen kommunikaation ja kielen kehityksen arviointimenetelmä. Jyväskylä: Jyväskylän yliopiston Lapsitutkimuskeskus ja Niilo Mäki instituutti.

[22] Schults A., Tulviste T. (2016). Composition of Estonian infants’ expressive lexicon according to the adaptation of CDI/Words and Gestures. First Lang.

[23] Kortesmaa M., Heimonen K., Merikoski H., Warma M.L., Varpela V. (2001). Reynellin kielellisen kehityksen testi. Reynell Developmental Language Scales III – RDLS III. Helsinki: Psykologien Kustannus Oy.

[24] Anderson N.J., Graham S.A., Prime H., Jenkins J.M., Madigan S. (2021). Linking quality and quantity of parental linguistic input to child language skills: a meta-analysis. Child Dev.

[25] Pancsofar N., Vernon-Feagans L. (2006). Mother and father language input to young children: contributions to later language development. J Appl Dev Psychol.

[26] Majorano M., Rainieri C., Corsano P. (2013). Parents’ child-directed communication and child language development: a longitudinal study with Italian toddlers. J Child Lang.

[27] Shapiro N.T., Hippe D.S., Ramírez N.F. (2021). How Chatty are Daddies? An Exploratory study of infants’ language environments. J Speech Lang Hear Res JSLHR.

[28] Abraham E., Feldman R. (2022). The neural Basis of human fatherhood: a Unique Biocultural Perspective on Plasticity of brain and Behavior. Clin Child Fam Psychol Rev.

[29] Preza T., Hadley P.A. (2024). Parent Responsivity, language input, and the development of Simple Sentences. J Child Lang.

[30] Coughlan S., Quigley J., Nixon E. (2024). Parent-infant conversations are differentially associated with the development of preterm- and term-born infants. J Exp Child Psychol.

[31] Lester B.M., Salisbury A.L., Hawes K., Dansereau L.M., Bigsby R., Laptook A. (2016). 18-Month follow-up of infants cared for in a single-family room neonatal intensive care Unit. J Pediatr.

[32] Vohr B., McGowan E., McKinley L., Tucker R., Keszler L., Alksninis B. (2017). Differential effects of the single-family room neonatal intensive care Unit on 18- to 24-Month Bayley scores of preterm infants. J Pediatr.

[33] Welch M.G., Barone J.L., Porges S.W., Hane A.A., Kwon K.Y., Ludwig R.J. (2020). Family nurture intervention in the NICU increases autonomic regulation in mothers and children at 4-5 years of age: follow-up results from a randomized controlled trial. PLoS One.

[34] Moe A.M., Kurilova J., Afzal A.R., Benzies K.M. (2022). Effects of Alberta family integrated care (FICare) on preterm infant development: two studies at 2 Months and between 6 and 24 Months corrected age. J Clin Med.

[35] Ahlqvist-Björkroth S., Feeley N., Alberts J., Montirosso R., Lehtonen L. (2024). Parenting interventions in neonatal intensive care units take different approaches. Acta Paediatr Oslo Nor.

[36] He F.B., Axelin A., Ahlqvist-Björkroth S., Raiskila S., Löyttyniemi E., Lehtonen L. (2021). Effectiveness of the close collaboration with parents intervention on parent-infant closeness in NICU. BMC Pediatr.

[37] Toivonen M., Lehtonen L., Löyttyniemi E., Ahlqvist-Björkroth S., Axelin A. (2020). Close Collaboration with Parents intervention improves family-centered care in different neonatal unit contexts: a pre-post study. Pediatr Res.

[38] Ahlqvist-Björkroth S., Axelin A., Lehtonen L. (2024). Close collaboration with parents-Implementation and effectiveness. Acta Paediatr Oslo Nor.

[39] Mayne J., McGowan E., Chiem A., Nwanne O., Tucker R., Vohr B. (2022). Randomised controlled trial of maternal infant-directed reading among hospitalised preterm infants. Acta Paediatr Oslo Nor.

