# Supplementary material for: Parents’ Speech in the NICU and Language Development of Very Preterm Children at 12 and 24 Months
Source: J Pediatr Clin Pract. 2025 Jun 12;17:200156. doi: 10.1016/j.jpedcp.2025.200156 (PMC12240094; doi:10.1016/j.jpedcp.2025.200156)
Supplement: Data Statement [file mmc1.docx]

Data Statement

Deidentified individual participant data will be made available in addition to study protocols, the statistical analysis plan, and the informed consent form. The data will be made available following publication to researchers who provide a methodologically sound proposal for using the data to achieve the goals of the approved proposal. Proposals should be submitted to the corresponding author at [anette.aija@utu.fi](mailto:anette.aija@utu.fi).
